# A micromachined electrochemical angular accelerometer with highly integrated sensitive microelectrodes

**DOI:** 10.1038/s41378-022-00418-7

**Published:** 2022-09-15

**Authors:** Tian Liang, Bowen Liu, Mingwei Chen, Yulan Lu, Jian Chen, Deyong Chen, Junbo Wang

**Affiliations:** 1 State Key Laboratory of Transducer Technology, Aerospace Information Research Institute, Chinese Academy of Sciences, Beijing, 100190 China; 2 School of Electronic, Electrical and Communication Engineering, University of Chinese Academy of Sciences, Beijing, 100049 China

**Keywords:** Electrical and electronic engineering, Sensors

## Abstract

This paper presents a micromachined electrochemical angular accelerometer with highly integrated sensitive microelectrodes. Theoretical analyses and numerical simulations were conducted to model the angular accelerometer with key geometrical parameters (e.g., electrode spacing, via spacing and via size) optimized. Highly integrated sensitive microelectrodes were manufactured based on microfabrication and assembled to form MEMS-based electrochemical angular accelerometers. Device characterization was conducted, locating a sensitivity of 80 V/(rad/s^2^), a bandwidth of 0.01–18 Hz and a noise level of 3.98 × 10^−8^ (rad/s^2^)/√Hz. In comparison to a previously reported electrochemical angular microaccelerometer, a significant improvement in sensitivity (80 V/(rad/s^2^) vs. 10 V/(rad/s^2^)) was achieved due to the new structure of sensitive microelectrodes. These results indicated the potential of the developed MEMS-based electrochemical angular accelerometer in seismology, including natural disaster monitoring and resource exploration.

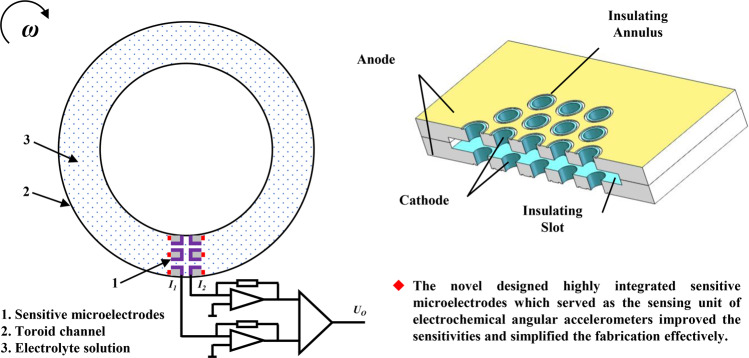

## Introduction

Many major earthquakes (e.g., near-field earthquakes) in history have records of rotational damages^1,2^, which proves that measurements of rotational motions are of great significance in the fields of seismology monitoring^1,3^. As the gold standard for detecting rotational motions in seismology, angular accelerometers can be mainly divided into two types based on solid or liquid inertial masses. Angular accelerometers based on solid inertial masses can be further divided into piezoelectric^4,5^, piezoresistive^6^, capacitive^2^ and electromagnetic^7,8^ principles, but they cannot be used in seismology monitoring, where high performances in the low-frequency domain are needed.

In contrast, angular accelerometers relying on liquid inertial masses feature excellent low-frequency performances because the velocities of ion movement in solution are extremely low in comparison with electron velocities^9^, which can be classified into molecular circular angular accelerometers and electrochemical angular accelerometers. Molecular circular angular accelerometers^10,11^ detected rotational signals relying on the interface effect of the electric double layer, which suffered from low sensitivities and complex manufacturing processes. Meanwhile, electrochemical angular accelerometers^12–18^ featured high sensitivities in the low-frequency domain, since oxidation–reduction reactions on sensitive electrodes were adopted as a transduction mechanism, which was suitable for applications in seismic explorations.

More specifically, Kozlov et al. reported the first electrochemical angular accelerometer^12^, which was then optimized through negative feedback^17^. However, the sensitive microelectrodes of these angular accelerometers were manufactured by ceramic technologies from platinum meshes, which suffer from complex fabrication processes and poor consistencies. Subsequently, Liu et al. developed MEMS-based electrochemical angular accelerometers^18,19^, which relied on integrated planar microelectrodes and suffered from low sensitivities due to limited electrode areas.

In this study, a micromachined electrochemical angular accelerometer with highly integrated sensitive microelectrodes was developed, where the electrode areas were significantly increased, leading to increases in device sensitivities. In addition, the fabrication process for the highly integrated sensitive microelectrodes was highly simplified, producing high consistencies in device fabrication. The following sections of the article include Materials and methods, Results and discussion, and a Conclusion.

## Materials and methods

### Structure and working principle

**Fig. 1:**
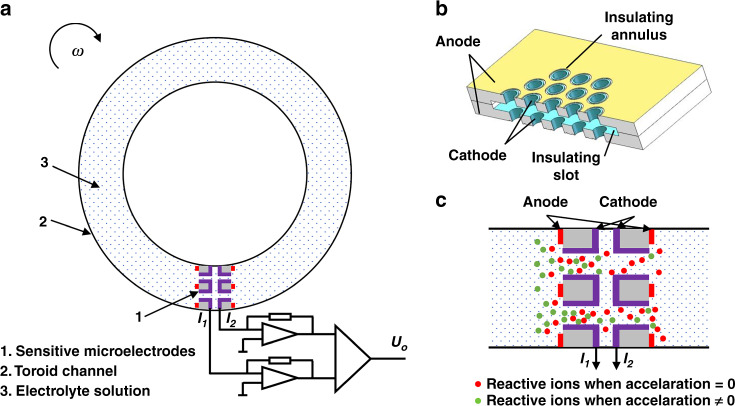
Structure and working principle of the micromachined electrochemical angular accelerometer with highly integrated sensitive microelectrodes. **a** Schematic of the electrochemical angular accelerometer mainly composed of highly integrated sensitive microelectrodes, a toroid channel and an electrolyte solution, where the structure of highly integrated sensitive microelectrodes was shown in **b**. **c** Working principle of the electrochemical angular accelerometer in which in response to an external angular acceleration, reactive ions in the electrolyte solution flow along the toroid channel and the vias on the sensitive microelectrodes. The corresponding electrochemical reactions occur on both sides of paired anodes and cathodes and converts angular acceleration into a voltage signal by a conversion circuit

The device structure of the developed electrochemical angular accelerometer mainly consisted of highly integrated sensitive microelectrodes (anode–cathode–cathode–anode), a toroid channel and an electrolyte solution made of KI and I_2_ (see Fig. 1a). The structure of the highly integrated sensitive microelectrodes was based on two silicon substrates with vias and sandwiched by an insulating slot, where the outer surfaces of the two wafers were anodes and the sidewalls of vias were cathodes; therefore, the areas of cathodes were effectively increased (see Fig. 1b).

A schematic of the working principle of the electrochemical angular accelerometer is shown in Fig. 1c, where redox reactions occurring on the surfaces of the anodes and cathodes were $3I^ - - 2e \to I_3^ -$ and $I_3^ - + 2e \to 3I^ -$, respectively. Under the condition of no angular acceleration, reactive ions ($I_3^ -$, red dots) formed a stable gradient distribution around the cathodes with identical currents generated and a zero output. When there was an external angular acceleration, a relative movement between the sensitive microelectrodes and the electrolyte solution resulted in concentration changes of reactive ions, leading to unidentical currents on the two cathodes and a voltage output.

### Theoretical analysis and numerical simulation

The energy conversion process of the electrochemical angular accelerometer, which converts relative movements between the electrolyte solution and sensitive microelectrodes to current outputs through electrochemical reactions on the electrodes, can be regarded as a low-pass link^16,18^, where increases in the frequency of the relative movement decrease the output current and thus the device sensitivity, and the output differential currents can be described by Faraday’s law:

1 $$I_o = 2F\left( {\mathop {\int}\limits_{S_1} {{{{\boldsymbol{J}}}} \cdot {{{\boldsymbol{n}}}}_{{{\boldsymbol{1}}}}ds - } \mathop {\int}\limits_{S_2} {{{{\boldsymbol{J}}}} \cdot {{{\boldsymbol{n}}}}_{{{\boldsymbol{2}}}}ds} } \right)$$

where *F* is the Faraday constant, *n*_*1*_ and *n*_*2*_ are the unit normal vectors of the two cathode surfaces, *J* is the ion flux of reactive ions on the cathode surface, and *S*_*1*_ and *S*_*2*_ are the areas of the cathodes, where the increase in cathode area (decrease via spacing) effectively improves device sensitivity.

The ion flux *J* can be described by the Nernst–Plank equation:

2 $${{{\boldsymbol{J}}}} = - D\nabla C - \frac{{zF}}{{RT}}DC\nabla \phi + C{{{\boldsymbol{V}}}}$$

where *D* is the diffusion coefficient, *C* is the ion concentration, *z* is the number of ion charges, *R* is the gas constant, *T* is the Kelvin temperature of the electrolyte solution, *ϕ* is the electric potential, and *V* is the velocity of the electrolyte solution near the sensitive microelectrodes.

The first and second items in Eq. (2) represent the diffusion process and the electromigration process, which were equal on the two cathodes, while the third item referred to the process of convection, causing the opposite change in ion flux on the two cathodes. Thus, the convection flux was determined by the concentration of reactive ions and the velocity of the electrolyte solution, which were affected by key parameters (electrode spacing and via size) of the sensitive microelectrodes.

However, theoretical analysis of the energy conversion process was complex, and thus, numerical simulations were conducted to determine key parameters of the sensitive microelectrodes based on a two-dimensional model. More specifically, physical fields of laminar flow and tertiary current distribution were adopted, where a body force in a sinusoidal mode standing for external acceleration was applied as the input signal and currents on cathodes were used as output signals.

**Fig. 2:**
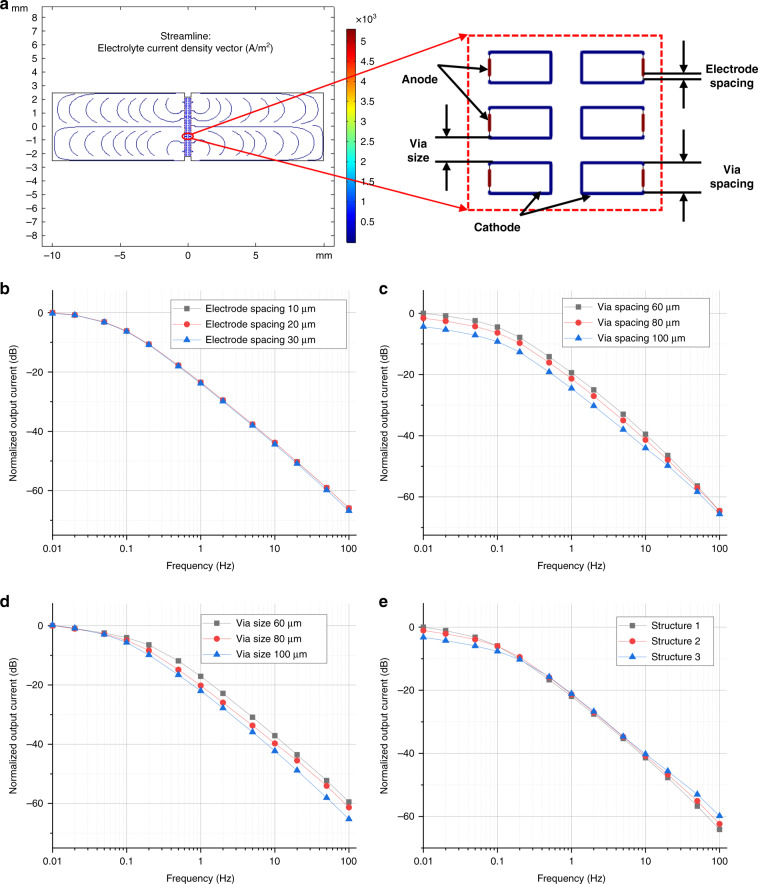
Numerical simulations of the micromachined electrochemical angular accelerometer with highly integrated sensitive microelectrodes. **a** Numerical simulations of the model demonstrating the current density streamlines of the electrolyte with the relationship between the frequency of the liquid acceleration and the output current of the electrodes modulated by key geometrical parameters of electrode spacing, via spacing, and via size were shown in **b**–**d**. **e** Relationships between the frequency of liquid acceleration and the output current of three types of proposed sensitive microelectrodes

Figure 2 shows the frequency responses of the electrochemical angular accelerometer with the highly integrated sensitive microelectrodes based on numerical simulations. Figure 2a shows the simulation results of the current density streamlines of the electrolyte solution, where the relationships between the frequency of input acceleration and the output current modulated by key geometrical parameters, including electrode spacing, via spacing and via size, are shown in Fig. 2b–d, respectively. According to these simulations, it was observed that the frequency increase of the input acceleration decreased the amplitudes of output currents, which functioned as a low-pass link. More specifically, (1) the decrease in electrode spacing demonstrated little effect on the output currents because the direction of electrolyte acceleration was perpendicular to the surfaces of the anodes; (2) the decrease in via spacing was shown to increase output currents at low frequencies due to increased cathode areas; and (3) the decrease in via size was shown to increase output currents at high frequencies because of the increasing hydrodynamic resistance. Based on these simulations, three types of sensitive microelectrodes with key geometrical parameters of via spacing of 60 μm and via size of 100 μm for structure 1, via spacing of 80 μm and via size of 80 μm for structure 2, and via spacing of 100 μm and via size of 60 μm for structure 3 were designed, and the relationships between the frequency of input acceleration and the output current are shown in Fig. 2e, where the increase of via spacing and decrease of via size can realize a wider raw 3 dB bandwidth.

### Fabrication

**Fig. 3:**
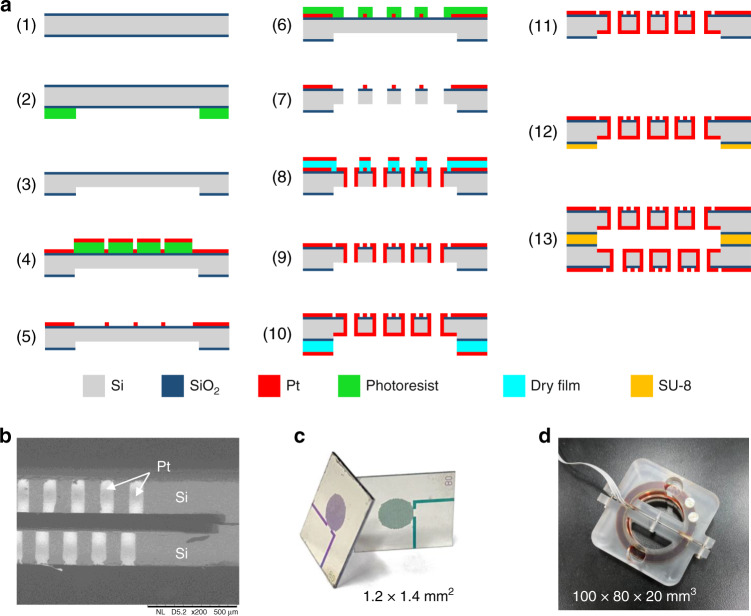
Fabrication and assembling of the micromachined electrochemical angular accelerometer. **a** The schematic of manufacturing process of the highly integrated sensitive microelectrodes as the key component of the micromachined electrochemical angular accelerometer includes key steps of (1) wafer cleaning, (2) lithography, (3) deep reactive ion etching, (4) lithography and sputtering, (5) lift-off, (6) lithography, (7) deep reactive ion etching, (8) lithography and sputtering, (9) lift-off, (10) lithography and sputtering, (11) lift-off, (12) SU-8 imprint, (13) bonding. **b** SEM of the cross-section view of the sensitive microelectrodes. **c** The fabricated highly integrated sensitive microelectrodes. **d** Prototype of the developed angular accelerometer based on the highly integrated sensitive microelectrodes

The MEMS-based manufacturing processes of the angular accelerometer with highly integrated sensitive microelectrodes are illustrated in Fig. 3a. Key steps included (1) wafer cleaning, (2) lithography to form patterns of the insulating slot, (3) deep reactive ion etching to form the insulating slot, (4) lithography and sputtering to form the anode, (5) lift-off for photoresist removal, (6) lithography to form the via patterns, (7) deep reactive ion etching to form vias, (8) lithography and sputtering to form the cathode, (9) lift-off to remove the dry film, (10) lithography and sputtering to connect the cathode together, (11) lift-off to remove the dry film, (12) SU-8 imprint through a wafer spin coated with SU-8, and (13) bonding.

Figure 3b shows a cross-section view of the sensitive microelectrodes based on SEM (scanning electron microscope), where the sidewalls were covered by platinum as cathodes. Figure 3c shows an image of the fabricated sensitive microelectrodes, which were assembled to form a prototype of the electrochemical angular accelerometer (see Fig. 3d).

## Results and discussion

### Sensitivity

The characterization of the developed micromachined electrochemical angular accelerometers was conducted by a national-standard angular vibration turntable at the Beijing Precision Engineering Institute for Aircraft Industry (BPEI), with amplitude (0–100°/s^2^) and frequency (0.01–25 Hz) under control.

**Fig. 4:**
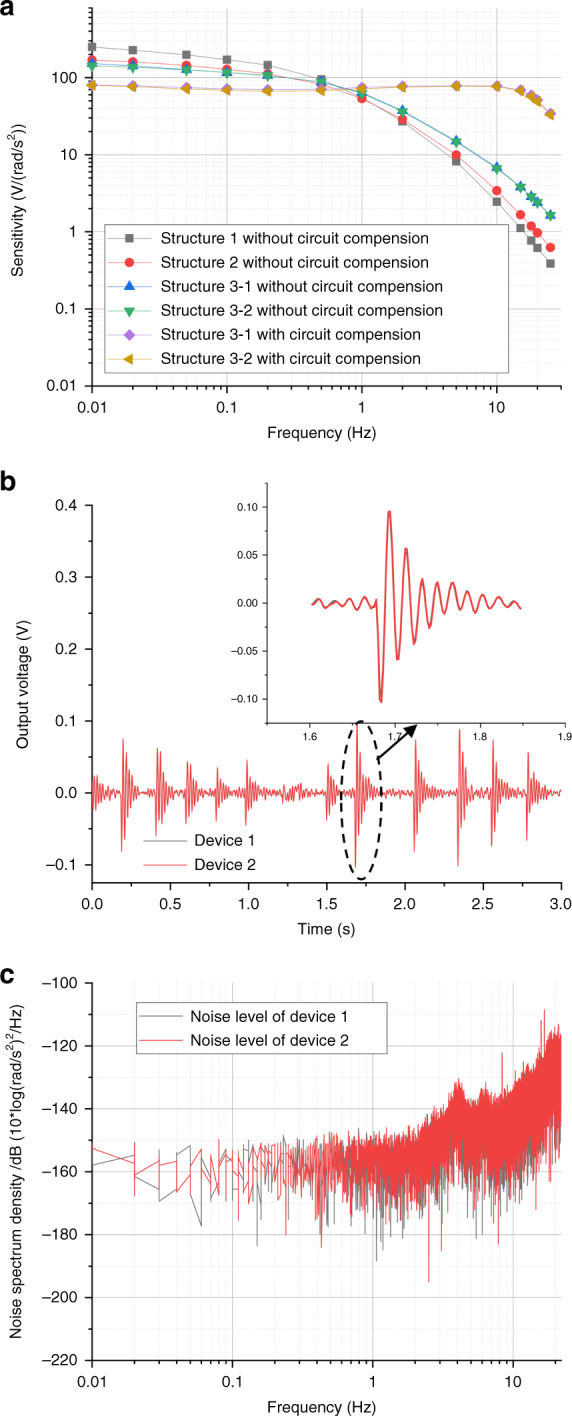
Characterization of the micromachined electrochemical angular accelerometer. **a** Sensitivity characterization of the developed electrochemical angular accelerometers with highly integrated sensitive microelectrodes where the horizontal axis represented the input frequency and the vertical axis represented sensitivity. **b** Consistency characterization of the developed electrochemical angular accelerometers where the horizontal axis represented time and the vertical axis represented output voltage. **c** Noise characterization of the developed electrochemical angular accelerometers where the horizontal axis represented the input frequency and the vertical axis represented power spectral density

Figure 4a shows the results of the sensitivity characterization of the developed electrochemical angular accelerometers, where the horizontal axis represents the input frequency (0.01–25 Hz), and the vertical axis represents the device sensitivity. More specifically, the aforementioned three types of sensitive microelectrodes were characterized.

As shown in Fig. 4a, when the spacing was reduced from 100 μm to 80 μm and 60 μm, the sensitivities of the electrochemical angular accelerometers at low frequencies increased from 152 V/(rad/s^2^) at 0.01 Hz to 168 V/(rad/s^2^) at 0.01 Hz and 249 V/(rad/s^2^) at 0.01 Hz. In addition, when the via size was reduced from 100 μm to 80 μm and 60 μm, the sensitivities of the electrochemical angular accelerometers at high frequencies increased from 0.38 V/(rad/s^2^) at 25 Hz to 0.62 V/(rad/s^2^) at 25 Hz and 1.38 V/(rad/s^2^) at 25 Hz.

These characterization results were consistent with numerical simulations where similar effects of via spacing and via size on device sensitivities were found. Among them, the 3 dB bandwidth of the three structures was quantified as 0.01–0.05 Hz, 0.01–0.1 Hz and 0.01–0.2 Hz, respectively. Since structure 3 with 100 μm for via spacing and 60 μm for via size demonstrated the largest bandwidth and the highest high-frequency sensitivities, it was chosen as the micromachined angular accelerometer with circuit compensations (a pole compensation circuit^20^ and a feedback circuit^19^), where the sensitivity and 3 dB bandwidth were quantified as 80 V/(rad/s^2^) and 0.01–18 Hz, respectively.

In comparison to previously reported electrochemical angular accelerometers relying on platinum mesh electrodes and planar microelectrodes, the developed electrochemical angular accelerometer with highly integrated sensitive microelectrodes featured a higher sensitivity (80 V/(rad/s^2^) vs. 8 V/(rad/s^2^) vs. 10 V/(rad/s^2^)) and a larger 3 dB bandwidth (0.01–18 Hz vs. 0.02–10 Hz vs. 0.01–8 Hz) (see Table 1).

**Table 1:** Comparison of key performances of angular accelerometers

Working principle	Ref	Sensitivity /V/(rad/s^2^)	Bandwidth (−3 dB) /Hz	Noise/(rad/s^2^)/Hz^1/2^
Capacitive	^2^	0.4~2.5	0.05–20	/
Electromagnetic	^7^	5~50	0.03–30	/
	^8^	1~10	0.7–30	/
Electrochemical	^17^	8	0.02–10	−105 dB
	^18^	10	0.01–8	−118 dB
	This study	80	0.01–18	−148 dB

### Consistency

In consistency characterization, two fabricated electrochemical angular accelerometers were placed together and sampled by the same data acquisition card under a few shocks. The recorded signals of the two devices are shown in Fig. 4b, where the horizontal axis represents time and the vertical axis represents the output voltage of the two devices. A high consistency with the cross-correlation coefficient between the two devices was quantified as 0.997.

### Noise Level

The characterization of the noise levels of two electrochemical angular accelerometers was carried out in a low-noise room and sampled at night. The noise characteristics were calculated with a correlation evaluation method^21^ based on two devices with high consistency. Figure 4c shows the characterization results of noise levels, where the horizontal axis represents the input frequency and the vertical axis represents the power spectrum density. The power spectrum density of the developed devices was shown to increase steadily with increasing frequency. More specifically, in comparison to the aforementioned counterparts, the developed electrochemical angular accelerometer with highly integrated sensitive microelectrodes featured a lower noise level (−148 dB vs. −105 dB vs. −118 dB) (see Table 1).

## Conclusion

In this study, a micromachined electrochemical angular accelerometer with highly integrated sensitive microelectrodes was designed, fabricated and characterized. Key geometrical parameters (electrode spacing, via spacing and via size) of the highly integrated sensitive microelectrodes were determined through theoretical analysis and numerical simulation. The developed electrochemical angular accelerometers were manufactured based on microfabrication, which featured high fabrication repeatability. Based on the results of performance characterization, key parameters (such as sensitivity, 3 dB bandwidth, consistency and noise level) of the developed electrochemical angular accelerometers were better than previously reported counterparts, illustrating a strong application potential in the fields of seismic detection and resource exploration.
